# In vitro effects of verteporfin on ocular cells

**Published:** 2013-02-20

**Authors:** David A. Ammar, Malik Y. Kahook

**Affiliations:** University of Colorado Hospital Eye Center, Department of Ophthalmology, University of Colorado Denver, Aurora, CO

## Abstract

**Purpose:**

Photodynamic therapy (PDT) laser light in conjunction with the benzoporphyrin derivative verteporfin is a current clinical treatment for choroidal vascular diseases such as age-related macular degeneration. The aim of this study was to examine the effects of PDT laser-activated and inactive verteporfin on various cultured ocular cells.

**Methods:**

Primary human scleral fibroblasts (hFibro), primary human trabecular meshwork (TM) cells (hTMC), primary porcine TM cells (pTMC), and a human retinal pigment epithelial cell line (ARPE-19 cells) were treated with verteporfin with and without activation by PDT laser. Cell viability was determined according to mitochondrial enzyme activity (3-(4,5- dimethyl-2-thiazoyl)-2,5-diphenyl-2H-tetrazolium bromide assay).

**Results:**

PDT laser treatment alone was insufficient to cause significant cell death in any of the cell types tested. Twenty-four-hour exposure to inactive verteporfin (without PDT laser) caused a dose-dependent decrease in cell viability in hFibro and hTMC, and to a lesser extent ARPE-19 cells. Verteporfin (0.5 µg/ml) without PDT laser activation caused a slight but statistically insignificant reduction in cell viability in hFibro (81.5%±19.3%), pTMC (82.9%±6.7%), hTMC (80.3%±7.7%), and ARPE-19 cells (84.5%±14.9%). Verteporfin (0.5 µg/ml) plus 50 µJ/cm^2^ PDT laser treatment significantly decreased viability in hFibro (13.5% ± 3.3%), pTMC (7.1%±1.5%), hTMC (11.1%±5.2%), and ARPE-19 (44.5%±7.8%). Similar results were obtained in cells where verteporfin incubation was followed by washout before PDT laser, indicating that verteporfin is internalized by the studied cell lines.

**Conclusions:**

PDT laser-induced cell death was obtained with coincubation of verteporfin or preincubation followed by washout. These results suggest a potential future use of PDT therapy for selective in vivo removal of targeted ocular cells beyond the current use for destroying vascular endothelial cells.

## Introduction

Age-related macular degeneration (AMD) is the leading cause of vision loss in patients over the age of 40, with the worst prognosis for patients with neovascular or wet AMD [1]. In this latter case, loss of vision occurs due to abnormal blood vessel growth originating from the choroidal vasculature. Photodynamic therapy (PDT) laser light in conjunction with the benzoporphyrin derivative verteporfin is a method approved by the U.S. Food and Drug Administration for treating choroidal vascular diseases of the eye. Following intravenous administration, activation of verteporfin by the PDT laser (about 688 nm) yields highly reactive oxygen radicals that damage the cells of the vasculature, resulting in localized vessel occlusion. Although several case reports of PDT therapy used to target neovascular diseases of the anterior chamber have been published [2-4], little is known of the effects of verteporfin-PDT therapy on tissues beyond the retina, retinal pigment epithelium (RPE), and vascular endothelium.

In the following experiments, we attempted to expand the laboratory knowledge of the effects of verteporfin on scleral fibroblasts and trabecular meshwork (TM) cells. We found that, under identical conditions, human scleral fibroblasts and TM cells are more sensitive to verteporfin-induced cell death than RPE cells. In this study, we describe how TM cells could be specifically targeted using PDT, potentially leading to new experimental models of ocular hypertension, or possibly a new therapeutic modality for treating glaucoma by inducing local remodeling in the outflow system of the eye.

## Methods

### Cell culture media and reagents

The Fibroblast Medium (FM, ScienCell Research Laboratories, Carlsbad, CA) consisted of a proprietary basal medium formulation supplemented with 2% fetal bovine serum (FBS), 1% fibroblast growth supplement, and 1% penicillin/streptomycin. Dulbecco’s modified Eagle Medium (DMEM), qualified FBS, penicillin-streptomycin (100× solution), and phosphate-buffered saline (PBS: 9 g/l sodium chloride, 0.795 g/l sodium phosphate dibasic heptahydrate, 0.144 g/l potassium phosphate monobasic) were purchased from Invitrogen/Life Technologies (Grand Island, NY). Rat tail type I collagen was purchased from Becton Dickson Biosciences (San Jose, CA). The metabolic activity indicator 3-(4,5- dimethyl-2-thiazoyl)-2,5-diphenyl-2H-tetrazolium bromide (MTT) was purchased from Sigma Aldrich (St. Louis, MO). Verteporfin (Visudyne, QLT Ophthalmics Inc., Menlo Park, CA) came as a lyophilized powder of 15 mg active ingredient in approximately 765 mg of inactive ingredients. Flat-bottom 96-well culture plates were obtained from Corning-Costar (Lowell, MA).

### Cell lines and establishment of primary cell cultures

ARPE-19, an RPE cell line spontaneously arising from a primary culture of human RPE cells, was purchased from American Type Culture Collection (Manassas, VA) and cultured according to the manufacturer’s instructions. Primary human trabecular meshwork cells (hTMC), isolated from the juxtacanalicular and corneoscleral regions of the human eye, were purchased from ScienCell Research Laboratories and cultured according to the manufacturer’s instructions. Primary human scleral fibroblasts (hFibro) were isolated from scleral strips taken from a normal donor eye (aged 92 years old) obtained from the San Diego Eye Bank (San Diego, CA). Approval was obtained from the Colorado Multiple Institutional Review Board for the use of human material, and the tenets of the Declaration of Helsinki were followed. The scleral strips were weighted down with sterile glass coverslips on a collagen-coated dish and maintained in DMEM containing 15% FBS and antibiotics for approximately 2 weeks. The resulting cells had a classic fibroblast morphology, and were passaged (1:3) into collagen-coated flasks and cultured in FM. Primary porcine trabecular meshwork cells (pTMC) were isolated from strips of porcine TM as described previously [5]. Briefly, tissue strips containing the TM region were dissected from the porcine eye, digested in collagenase type IV, and plated onto gelatin coated dishes. The pTMC were passaged (1:3) into collagen-coated flasks and cultured in FM.

### Cell culture conditions

The ARPE-19 cells used in these experiments were from the 20^th^ or 21^st^ passage and were cultured in DMEM containing 10% FBS and antibiotics. Approximately 1×10^4^ ARPE-19 cells were plated into uncoated 96-well plates 2–3 days before each experiment. The hTMC used in these experiments were from the fourth or fifth passage and were cultured in FM. Approximately 5×10^3^ hTMC were plated into collagen-coated 96-well plates 2–3 days before each experiment. The hFibro used in these experiments were from the eighth or ninth passage and were cultured in FM. Approximately 7.5×10^3^ hFibro were plated into collagen-coated 96-well plates 2–3 days before each experiment. The pTMC used in these experiments were from the fourth or fifth passage and were cultured in FM. Approximately 2.5×10^3^ pTMC were plated into collagen-coated 96-well plates 2–3 days before each experiment. The hTMC were cultured on collagen-coated tissue culture dishes and wells in FM.

### Verteporfin treatment of cultured cells

Verteporfin studies were initiated when the cultured cells reached >95% confluence, usually 2–3 days post plating. Experiments were performed in duplicate. For the initial toxicity studies, a range of verteporfin (0, 0.25, 1, 4, 10, and 25 µg/ml) dissolved in 150 µl of the appropriate culture medium was added to hFibro, hTMC, and ARPE-19 cells. The 96-well plates were shielded from light and incubated in a humidified incubator at 37 °C and 5% CO_2_ for 24 h. After the media containing verteporfin were removed, metabolic activity (MTT assay) was assayed without exposure to photodynamic therapy (PDT) laser light. For all subsequent toxicity studies of light-activated verteporfin, we used a single concentration of verteporfin (0.5 µg/ml) dissolved in 150 µl of the appropriate culture medium. The hFibro, hTMC, and ARPE-19 cells were exposed to PDT laser under two conditions. For the pretreat condition, cells were exposed to verteporfin for 24 h as above, washed twice in PBS, and cultured in verteporfin-free media for 3 h before being exposed to the PDT laser. For the cotreat condition, cells were exposed to verteporfin for 3 h and then exposed to the PDT laser without a change of the culture media. The beam of the PDT laser (VisuLas 690S laser; Carl Zeiss Meditec AG, Berlin, Germany) was centered at 688 nm, with more than 90% of its energy between 686 and 690 nm. The laser spot-size of the PDT laser was adjusted to encompass the entire 0.32 cm^2^ bottom area of a 96-well culture well. Cultured cells were exposed to 0, 50, or 100 µJ/cm^2^ of PDT laser followed by metabolic activity measurements (MTT assay).

### Cell viability/metabolic assays

The mitochondrial activity of the cultured cells was determined with MTT assay. Following the verteporfin experiments, the media were aspirated, and the cells were incubated in 100 µL of 0.5 mg/mL MTT dissolved in the appropriate culture media for 1 h at 37 °C. The MTT media were aspirated, and the purple formazan reaction product was solubilized by adding 100 µL dimethyl sulfoxide (DMSO). The absorbance of each sample was read at 540 nm in a Synergy 4 Multi-Mode Microplate Reader using the Gen5 Reader Control and Data Analysis Software (BioTek, Winooski, VT). After the background (A_540_ of DMSO) was subtracted, the data were normalized to the MTT absorbance of the appropriate untreated cell type (100% Live).

### Statistical analysis

Experiments were performed in duplicate. Mean values for each experimental condition were analyzed with the Student *t* test (Excel, Microsoft, Redmond WA); the level of significance was set at 0.05. Data are reported as the mean ± standard deviation (SD) of n=4 replicates.

## Results

### Dose-dependent toxicity of inactive verteporfin

**Figure 1:**
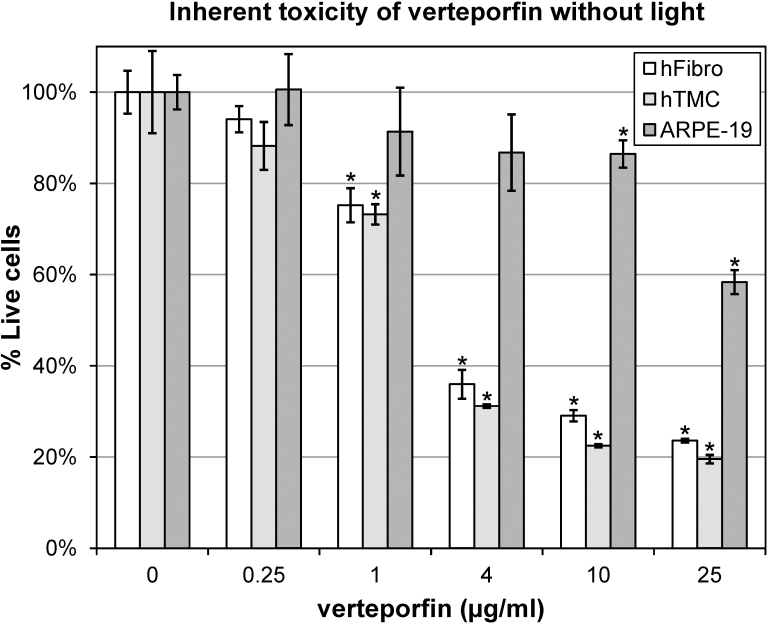
Viability of cultured ocular cells exposed to a range of verteporfin for 24 h without exposure to laser light. The percent of live cells was determined with 3-(4,5- dimethyl-2-thiazoyl)-2,5-diphenyl-2H-tetrazolium bromide (MTT) assay in human scleral fibroblasts (hFibro), human trabecular meshwork cells (hTMC), and a human retinal pigment epithelial cell line (ARPE-19). Data were normalized to untreated cells (0 µg/ml verteporfin; 100% Live), and plotted as the mean (n=4) with error bars representing the standard deviation. *=p<0.05 versus 0 µg/ml verteporfin treatment.

A range (0–25 µg/ml) of verteporfin was diluted in the appropriate cell culture media and then added to cultured ocular cells. Cells were protected from light and incubated for 24 h at 37 °C in a humidified CO_2_ incubator. Mitochondrial enzyme activity, determined with MTT assay, was used as a surrogate for cell viability (Figure 1). Increasing amounts of verteporfin without PDT laser activation (inactive verteporfin) was inherently toxic to the hFibro, hTMC, and ARPE-19 cells. There was no significant (p>0.05) decrease in cell viability at 0.25 µg/ml verteporfin in either the hFibro (94.1% ± 2.9%) or the hTMC (88.2%±5.2%) compared to the untreated hFibro (100.0%±4.7%) or the hTMC (100.0%±9.0%). Inactive verteporfin showed a dose-dependent increase in toxicity for the hFibro and hTMC at 1 µg/ml and above (p<0.05). Compared to the control hFibro (0 µg/ml verteporfin), cell viability decreased to 75.2%±3.7% (1 µg/ml), 35.9%±3.2% (4 µg/ml), 29.1%±1.2% (10 µg/ml), and finally 23.6%±0.4% (25 µg/ml). In the hTMC, viability decreased significantly to 73.2% ± 2.2% (1 µg/ml), 31.2%±0.4% (4 µg/ml), 22.5%±0.4% (10 µg/ml), and 19.6%±0.9% (25 µg/ml).

Inactive verteporfin was less toxic to ARPE-19 cells, with no statistically significant decrease (p>0.05) in cell viability between the untreated controls (100.0% ± 3.8%) and the cells treated with 0.25 µg/ml (100.6% ± 7.8), 1 µg/ml (91.4% ± 9.6), or 4 µg/ml (86.7% ± 8.4%). A small but significant decrease in cell viability occurred in the ARPE-19 cells treated for 24 h with 10 µg/ml verteporfin (86.5% ± 3.0%), which decreased to 58.4% ± 2.6% with 25 µg/ml verteporfin.

### Light activation of verteporfin increases its toxicity to cultured ocular cells

**Figure 2:**
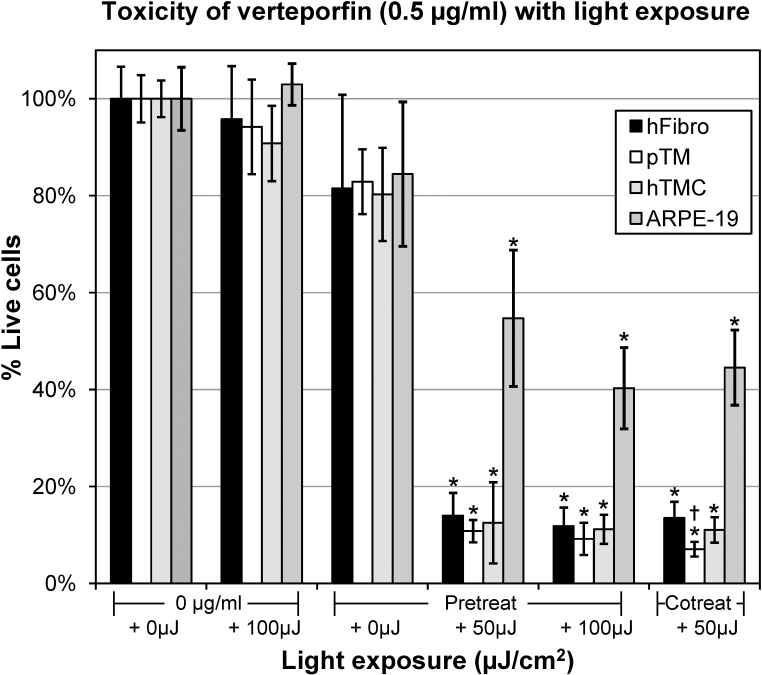
Viability of cultured ocular cells exposed to 0.5 µg/ml verteporfin with different intensities of laser light. The percent of live cells was determined with 3-(4,5- dimethyl-2-thiazoyl)-2,5-diphenyl-2H-tetrazolium bromide (MTT) assay in human scleral fibroblasts (hFibro), pig trabecular meshwork cells (pTMC), human trabecular meshwork cells (hTMC), and a human retinal pigment epithelial cell line (ARPE-19). Data were normalized to cells not treated with either verteporfin or laser light (0 µg/ml + 0 µJ/cm^2^; 100% Live), and plotted as the mean (n=4) with error bars representing the standard deviation. *=p<0.05 versus both 0 µg/ml + 0 µJ/cm^2^ and pretreat + 50 µJ/cm^2^.

To assess the relative toxicity of light-activated verteporfin, cultured hFibro, pTMC, hTMC, and ARPE-19 cells were incubated with 0.5 µg/ml of verteporfin at 37 °C in a humidified CO_2_ incubator. After 3 h, the cells in the verteporfin-containing media were exposed to 50 µJ/cm^2^ of the PDT laser and MTT assays were immediately performed (cotreat condition, Figure 2). In the absence of verteporfin, no statistically significant loss of cell viability was seen in any cultured cells with exposure of up to 100 µJ/cm^2^ PDT light (0 µg/ml + 100 µJ/cm^2^, Figure 2). Twenty-four hour incubation with 0.5 µg/ml inactive verteporfin caused a statistically insignificant reduction in cell viability in the hFibro (81.5%±19.3%), pTMC (82.9%±6.7%), hTMC (80.3%±7.7%), and ARPE-19 cells (84.5%±14.9%). However, 0.5 µg/ml verteporfin with the addition of 50 µJ/cm^2^ PDT light reduced the viability of the hFibro to 13.5%±3.3%, the pTMC to 7.1%±1.5%, the hTMC to 11.1%±5.2%, and the ARPE-19 cells to 44.5%±7.8% of the control levels (cotreat + 50 µJ/cm^2^, Figure 2).

### Verteporfin retains its cytotoxic effects with pretreatment only

Cultured hFibro, pTMC, hTMC, and ARPE-19 cells were incubated with 0.5 µg/ml of verteporfin at 37 °C in a humidified CO_2_ incubator. After 24 h, verteporfin-containing media were removed, and the cells were washed twice and then incubated in fresh cell culture media for 3 h. The cells were then exposed to 50 µJ/cm^2^ and 100 µJ/cm^2^ of laser, and MTT assays were immediately performed (pretreat condition, Figure 2).

The hFibro pretreated for 24 h with 0.5 µg/ml verteporfin underwent in a slight but not significant drop in cell viability (81.5%±19.3% live) compared to the untreated controls (100.0%±6.6%). When the hFibro were exposed to the 50 µJ/cm^2^ PDT laser, viability decreased to 14.0% ± 4.7% live cells. The viability of the pTMC pretreated with 0.5 µg/ml verteporfin (82.9%±6.7% live) was not significantly different from the viability of the untreated controls (100.0%±4.9%). This viability decreased to 10.8% ± 2.3% live cells when the pTMC were exposed to the 50 µJ/cm^2^ PDT laser. The hTMC pretreated with 0.5 µg/ml verteporfin underwent in a slight but not significant drop in cell viability (80.3%±7.7% live) compared to the untreated controls (100.0% ± 3.5%). When the hTMC were exposed to the 50 µJ/cm^2^ PDT laser, viability decreased to 12.5%±1.9% live cells. The viability of the ARPE-19 cells pretreated with 0.5 µg/ml verteporfin (84.5%±14.9% live) was not significantly different from the viability of the untreated controls (100.0%±6.5%). This viability decreased to 54.7%±14.1% live cells when the 50 µJ/cm^2^ PDT laser was added. In all cells (hFibro, pTMC, hTMC, and ARPE-19) pretreated with verteporfin, increasing the PDT laser exposure from 50 µJ/cm^2^ to 100 µJ/cm^2^ did not result in any further significant decrease in cell viability (p>0.05).

In addition, we found no statistically significant difference in the cell viability of cells treated with verteporfin (0.5 µg/ml) plus PDT laser between the pretreat or cotreat condition in the hFibro, hTMC, and ARPE-19 cells. There was an extremely small but statistically significant difference between the pretreat and cotreat conditions in the pTMC (10.8%±2.3% versus 7.1%±1.5%).

## DISCUSSION

Intravenously (IV) administered verteporfin has been used previously to target blood vessels within the drainage angle to treat neovascular glaucoma, to target iris vessels for treating pseudoexfoliation glaucoma, and to target ciliary body vasculature to reduce aqueous humor production [2-4]. In a rabbit model of glaucoma filtration surgery, PDT has shown promise in reducing bleb failure by decreasing fibrosis presumably by targeting local neovascularization [6]. Administering verteporfin IV led to rapid (about 5 min) accumulation of drug in the choroid, RPE, and ciliary body followed quickly by accumulation in the photoreceptors [7]. No appreciable amount of verteporfin was detected in corneal tissue, indicating that the blood–eye barrier prevents the IV-administrated agent from entering the aqueous and vitreous spaces. Perhaps due to this partitioning, we know of no previous attempt to determine the effects of verteporfin on intraocular cell types that reside beyond the blood–eye barrier.

In this work, we demonstrate the relative toxicity of inactive and light-activated verteporfin in scleral fibroblasts, trabecular meshwork cells, and retinal pigment epithelial cells. We determined that the fibroblasts and trabecular meshwork cells were more sensitive to inactive and light-activated verteporfin than the ARPE-19 cells. Similarly, we found that in the absence of verteporfin, cultured cells exposed to PDT laser therapy at twice the recommended treatment protocol (2×50 µJ/cm^2^) for neovascular AMD exhibited no detrimental effect. However, in the presence of verteporfin, we found that a single standard treatment protocol (50 µJ/cm^2^) was sufficient to fully activate all verteporfin within the focal area, since treatment with 100 µJ/cm^2^ caused no further cell death. Approximately 80% of human fibroblasts and TM cells (human and porcine) were killed when exposed to 0.5 µg/ml verteporfin plus PDT laser, while under these conditions only approximately 50%–60% of RPE cells were killed. In this work, we did not attempt to ascertain the lowest threshold of PDT laser-activated verteporfin needed to kill fibroblasts or TM cells. Work by others indicates that in leucocytes light-activated verteporfin has an medial lethal dose (LD_50_) in the range of 0.01 to 0.02 µg/ml [8,9]. Prior work in leukocytes has shown no toxicity in the absence of light [8]. Our work does however help determine levels of verteporfin that may be safe for use after direct injection in the eye.

We have shown that fibroblasts and TM cells are more sensitive to increasing concentrations of inactive verteporfin than ARPE-19 cells. In the absence of the PDT laser, there was no statistically significant decrease in viability with 0.5 µg/ml of verteporfin. We noted significant toxicity in hFibro and hTMC (but not ARPE-19 cells) when the amount of verteporfin was increased to 1 µg/ml and above. If IV-administered verteporfin has a maximum blood plasma concentration of 2 µg/ml and a clearance half-life of about 5–6 h (Visudyne Food and Drug Administration label), plasma levels should drop below 1 µg/ml within 6 h. Therefore, this inherent toxicity to certain cell types may be less relevant in vivo when verteporfin is administered by IV, but must be considered if it is administered by intravitreal or intracameral injection. We believe that the data presented here support the hypothesis that an intraocular injection of ≤0.5 µg/ml could allow for PDT-laser killing of TM cells and scleral fibroblasts without having toxic effects in other tissues not within the focal beam of the laser.

Our data also suggest that cultured cells can internalize inactive verteporfin, as shown in the pretreat experiments in Figure 2. In these experiments, cells were exposed to verteporfin, washed, and then incubated in verteporfin-free media. After the cells were exposed to the PDT laser, the percentage of live cells in the pretreat conditions was nearly identical to the percentage of live cells in the cotreat conditions. The simplest explanation suggests that verteporfin is taken up by these cells during the 24 h pretreatment, although future experiments need to be performed to verify internal accumulation. This accumulation of verteporfin within cells has been previously demonstrated in other cell types, and the internalization process depends on binding and internalizing verteporfin via low-density lipoprotein (LDL) receptors [10]. LDL receptors have been functionally shown to be present on TM cells [11], normal fibroblasts [10], and RPE [12]. The internalization of verteporfin by cells expressing LDL receptors (TM and fibroblasts) is also a potential mechanism that should prove useful in future studies involving PDT therapy.

In conclusion, we have shown that TM cells were more susceptible to verteporfin than RPE cells, and we hypothesize that TM cells internalize more verteporfin than RPE cells because of the ability of LDL receptors to internalize verteporfin. Taking advantage of this difference, our findings show that PDT can be used for targeted killing of TM cells that could lead to new experimental models of ocular hypertension. Alternatively, targeted anterior segment PDT therapy may be a potential glaucoma treatment, in which selective destruction of TM cells in certain regions of the meshwork may lead to localized tissue remodeling and a subsequent increase aqueous outflow. Additionally, refinement of an ophthalmic gonioscopic lens that can deliver targeted light to the angle after verteporfin is injected in vivo will be necessary to minimize collateral damage. Selective killing of aqueous outflow system cells could also allow for future stem cell therapies designed to repopulate the drainage system with appropriately functioning cells. The use of direct intraocular injections of verteporfin in future in vivo experiments will be useful in assessing the translational utility of PDT therapy in treating diseases of the outflow system of the eye.
